# Therapeutic prospect on umbilical cord mesenchymal stem cells in animal model with primary ovarian insufficiency: a meta-analysis

**DOI:** 10.3389/fmed.2023.1211070

**Published:** 2023-05-31

**Authors:** Xinrun Wang, Tianye Li, Xuechai Bai, Yun Zhu, Meiliang Zhang, Liang Wang

**Affiliations:** ^1^Department of Gynecology, The Second Affiliated Hospital, Zhejiang University School of Medicine, Hangzhou, Zhejiang, China; ^2^Center for Reproductive Medicine, Department of Gynecology, Zhejiang Provincial People's Hospital (Affiliated People's Hospital, Hangzhou Medical College), Hangzhou, Zhejiang, China; ^3^Center for Clinical Big Data and Analytics, The Second Affiliated Hospital Zhejiang University School of Medicine, Hangzhou, Zhejiang, China; ^4^Department of Obstetrics and Gynecology, Yiwu Maternity and Children Hosptial, Yiwu Branch of Children's Hospital Zhejiang University School of Medicine, Yiwu, Zhejiang, China

**Keywords:** primary ovarian insufficiency, human umbilical cord mesenchymal stem cells, animal model, meta-analysis, estrous cycle, hormone level, folliculogenesis

## Abstract

**Background:**

Primary ovarian insufficiency (POI) leads to not only infertile but several adverse health events to women. Traditional treatment methods have their own set of limitations and drawbacks that vary in degree. Application of human umbilical cord mesenchymal stem cell (hUCMSC) is a promising strategy for POI. However, there is a lack of literatures on application of hUCMSC in human. Animal experimental model, however, can reflect the potential effectiveness of this employment. This study aimed to evaluate the curative effect of hUCMSC on animals with POI on a larger scale.

**Methods:**

To gather data, Pubmed, Embase, and Cochrane Library were searched for studies published up to April 2022. Various indices, including the animals' estrous cycle, serum sex hormone levels, and follicle number in the ovary, were compared between the experimental group and those with Premature Ovarian Insufficiency (POI).

**Results:**

The administration of human umbilical cord-derived mesenchymal stem cells (hUCMSC) has been shown to significantly improve the estrous cycle (RR: 3.32, 95% CI: [1.80, 6.12], *I*^2^ = 0%, *P* = 0.0001), but robustly decrease its length (SMD: −1.97, 95% CI: [−2.58, −1.36], *I*^2^ = 0%, *P* < 0.00001). It can also strikingly increase levels of serum estradiol (SMD: 5.34, 95% CI: [3.11, 7.57], *I*^2^ = 93%, *P* < 0.00001) and anti-müllerian hormone (SMD: 1.92, 95% CI: [0.60, 3.25], *I*^2^ = 68%, *P* = 0.004). Besides, it lowers levels of serum follicle-stimulating hormone (SMD: −3.02, 95% CI: [−4.88, −1.16], *I*^2^ = 93%, *P* = 0.001) and luteinising hormone (SMD: −2.22, 95% CI: [−3.67, −0.76], *I*^2^ = 78%, *P* = 0.003), and thus collectively promotes folliculogenesis (SMD: 4.90, 95% CI: [3.92, 5.88], *I*^2^ = 0%, *P* < 0.00001).

**Conclusions:**

Based on the presented findings, it is concluded that the administration of hUCMSC in animal models with POI can result in significant improvements in several key indicators, including estrous cycle recovery, hormone level modulation, and promotion of folliculogenesis. These positive outcomes suggest that hUCMSC may have potential as a treatment for POI in humans. However, further research is needed to establish the safety and efficacy of hUCMSC in humans before their clinical application.

**Systematic review registration:**

https://inplasy.com/inplasy-2023-5-0075/, identifier: INPLASY202350075.

## 1. Introductions

Primary ovarian insufficiency (POI), also known as premature ovarian failure (POF), is a syndrome characterized by reduced or absent ovarian function (hypogonadism) and elevated levels of gonadotropins, specifically luteinising hormone (LH) and follicle-stimulating hormone (FSH) (hypergonadotropic) (1, 2). This occurs due to the lack of negative sex-steroid and inhibin feedback. Therefore, POI is also referred to as hypergonadotropic hypogonadism. The condition is diagnosed when oocytes and the surrounding support cells are lost before the age of 40 years, along with serum FSH levels above the threshold range of 30–40 mIU/mL twice (at least 1 month apart). POI is a systemic disease that can lead to various effects. Recent research has summarized the long-term health consequences of POI, including an increased risk of cardiovascular disease (CVD), decreased bone mineral density, significantly reduced fertility, psychological distress, vulvovaginal atrophy, neurological effects, and overall reduced life expectancy (3). While the incidence of POI is not peculiar, the underlying causes of this condition remain largely unknown (4). Despite extensive research, the etiology of POI is still not fully elucidated, and it is considered a complex and multifactorial condition. Genetic disorders, such as chromosomal abnormalities, are among the most prevalent causes of POI (5). These disorders can lead to early ovarian failure and an increased risk of POI. However, other factors like autoimmune diseases, iatrogenic injuries, and infectious diseases can also contribute to the onset of POI (6–8). In some cases, autoimmune disorders like systemic lupus erythematosus or Hashimoto's thyroiditis can trigger the body's immune system to attack ovarian tissue, leading to POI (9). Additionally, with the increasement of gynaecologic cancer, medical treatments like chemotherapy, radiation therapy, or surgical removal of the ovaries can also cause damage to the ovarian tissue, leading to POI (10). Infections, such as mumps, tuberculosis, or sexually transmitted diseases like gonorrhea, can also contribute to POI by damaging the ovaries or disrupting their function (11). Given the complex and multifactorial nature of POI, early detection and timely intervention are crucial to help manage the condition and improve the quality of life of affected individuals. Therefore, a better understanding of the factors contributing to POI and advancements in diagnostic methods can aid in developing effective treatments and management strategies for this condition (12). Currently, traditional therapy for POI is limited. To patients without desire for pregnancy, hormone replacement therapy (HRT) is appropriate. HRT can significantly relieve POI symptoms and decrease bone fracture and CVD risks. It can even help fertility for those females who still have ovarian follicle reserve (13). Infertility treatment is another therapeutic aspect for POI. Oocyte donation is a traditional but useful way to help delivery, but is limited in many countries and regions. A way to preserve fertility is the cryopreservation of oocytes, embryos and ovarian tissues. For those who undergo radiotherapy, GnRH analog can help protect fertility, but some data are conflicting. Furthermore, a new method called *in vitro* activation (IVA) of dormant follicles can help patients with POI conceive as well (14). However, all of these therapies can be conducive to helping a small proportion of patients with POI. Human umbilical cord mesenchymal stem cell (hUCMSC) is mesenchymal stem cells derived from Wharton's jelly of a fetal umbilical cord. These cells have multiple differentiation potentials. They can generate cell types such as adipocytes, osteocytes and cartilage. In addition, neurons, astrocytes, glial cells, liver and islet cells are the potential lineage of hUCMSC (15). Stem cell therapy has been proposed for a long time. Some clinical trials have tried to understand the therapeutic effect of hUCMSC in POI. Evidence revealed follicular activated, estradiol (E_2_) increased and FSH decreased after hUCMSC transplantation in patients with POI (16, 17). Collagen scaffold with hUCMSC is another stem cell delivery approach that has shown a therapeutic effect. In an *in vivo* study, hUCMSC activated primordial follicles by phosphorylating FOXO3a and FOXO1 (17). Apart from clinical trials, many studies tested the therapeutic effect of hUCMSC on the ovary of animals. For instance, hUCMSC introduction led to an atretic follicle decrease and a healthy antral follicle increase in mice. Granulosa cell (GC) apoptosis induced by POI was inhibited. Based on molecular analysis, the expression of SOD2, CAT and Bcl2 mRNA increased, whereas *Bax* mRNA expression declined (18). Given that these genes are associated with oxidation and apoptosis, hUCMSC infusion may influence the antioxidative and antiapoptotic procedures of the ovary. Furthermore, *in vivo* cell culture found that hUCMSC can secrete VEGF, IGF-1 and HGF (19). Through Sirius red and Masson trichrome staining of the ovary tissue, researchers found that fibrosis developed in POI rats, but after hUCMSC treatment, the fibrosis area was significantly reduced. The TGF-β_1_ signaling pathway is a crucial immune regulative factors (20), also reportedly involved in hUCMSC regulation. The hUCMSC can inhibit the expression of TGF-β_1_ and p-smad3 in the ovary, thereby depressing the differentiation of stromal cells into inner theca cells (TCs) and consequently inhibiting fibrosis in POI rats (21). However, only few integrated analyses have been found. Thus, this study aimed to summarize the results of animal studies investigating on hUCMSC and POI, form more valid evidence and confirm the therapeutic effect of hUCMSC on experimental animals compared with the POI model by analyzing the estrous cycle, serum sex hormone and ovarian follicles in the two groups.

## 2. Methods

This systematic meta-analysis appraises the association between employment of hUCMSC and the indices of ovarian reserve function in experimental animal models. We followed the preferred reporting items for meta-analysis and systematic review (PRISMA) 2020 guidelines and putting forward the research question using the PECOS format. We have registered at International Platform of Registered Systematic Review and Meta-analysis Protocols (INPLASY). Registration number is INPLASY202350075.

### 2.1. Search strategy

We searched the Pubmed, Embase and Cochrane Library databases. Specific search strategy is “((Primary Ovarian Insufficiency) OR (Premature Ovarian Failure) OR (Gonadotropin-Resistant Ovary Syndrome) OR (Hypergonadotropic Ovarian Failure)) AND ((Stem cell) OR (Progenitor Cell))”. To conclude, we used MeSH terms and their typical synonyms and combined them with “OR.” Then, we combined the results of “primary ovarian insufficiency” and “stem cell” with “AND.” All results from the date of database establishment to 1 April 2022 were included.

### 2.2. Inclusion and exclusion criteria

Initially, we excluded all duplicated studies. Subsequently, we collected studies that met the following criteria: female animals; hUCMSC; successful POF model establishment; and serum hormone, follicle count and estrous cycle as the outcome. Furthermore, the following five study types were excluded: reviews and meta-analysis, studies that are not associated with stem cell or POI, non-animal studies, case reports and animal studies without hUCMSC application. After selecting studies related to hUCMSC and POI, we thoroughly read the full text and further excluded studies that we failed to collect the exact data and studies with no outcomes that we aimed.

### 2.3. Data extraction and statistical analysis

Data were extracted and qualifiedly assessed by using “SYRCLE animal experiment bias risk assessment form.” We used risk ratios (RRs) with 95% confidence intervals (CI) for categorical data, and standardized mean difference (SMD) for numerical data to combine studies. If the heterogeneity test showed *I*^2^ > 50%, we used random effects model. Otherwise, we used fixed effects model. All statistical data were analyzed on RevMan 5.4 (22). The extracted data from each study included the first author, country or region, publishing year, experiment animal, POI model establishing method, hUCMSC intervention situation, group situation and outcome data. During the analysis, we firstly tested the heterogeneity of the studies and selected the effects model, as mentioned before. Then, we divided the studies according to unit, injection location, hUCMSC concentration, transplantation time and follicle type for the subgroup analyses. Sensitivity was assessed by eliminating studies one by one. We also used funnel plot to determine the publication bias. All statistical significances were defined at *P* < 0.05.

## 3. Results

### 3.1. Description of search results

**Figure 1:**
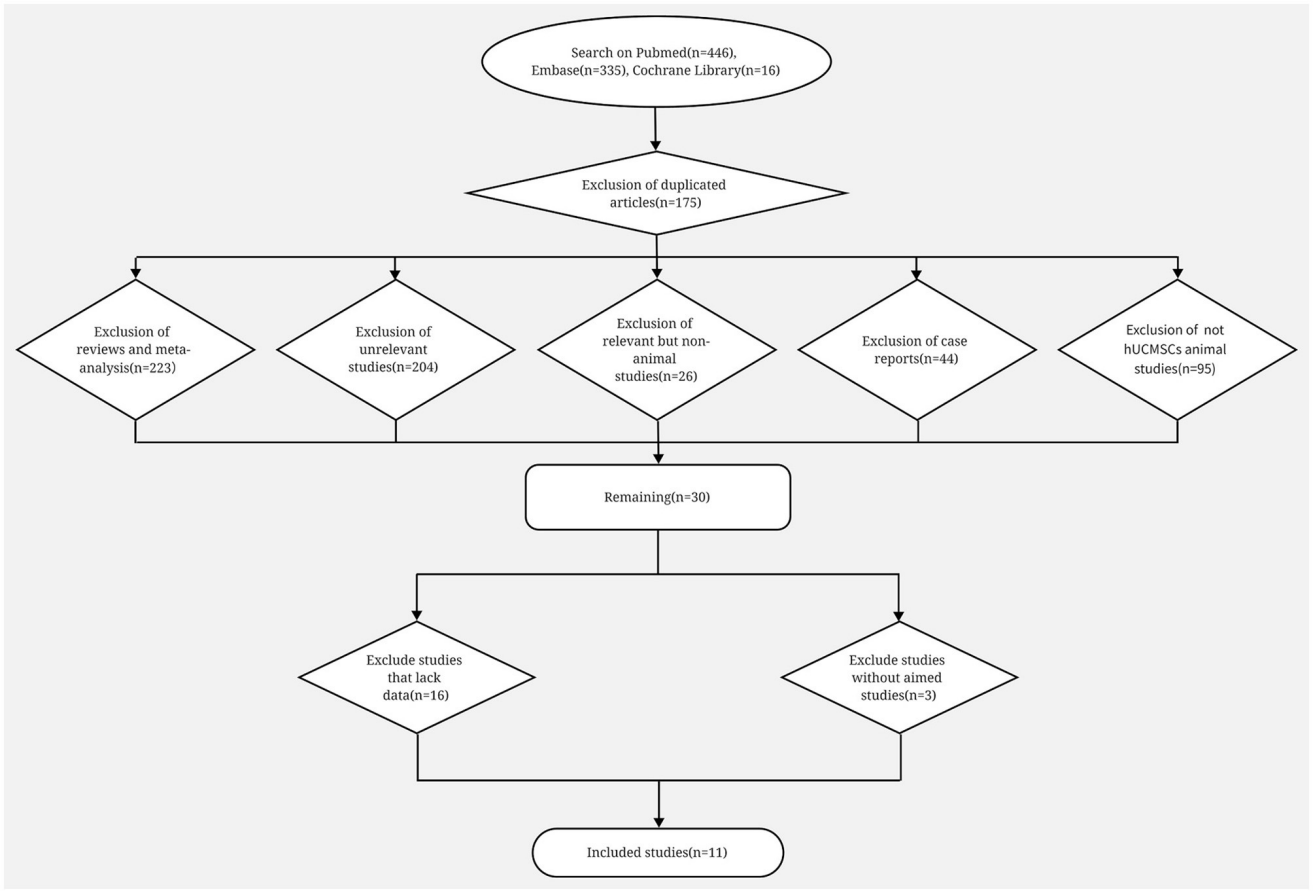
Flow diagram on search procedure of Pubmed, Embase and Cochrane Library and the exclusion criteria.

We identified 446, 335 and 16 studies from Pubmed, Embase and Cochrane Library, respectively. Among them, 175 duplicate studies, 223 reviews or meta-analyses, 204 studies that are not associated with stem cell or POI, 26 animal studies, 44 case reports and 95 animal studies without hUCMSC used were excluded. Conclusively, 30 animal studies remained, and they were all correlated to hUCMSC and POI. After full text reading, we further excluded 16 studies because we failed to obtain the exact data, and three studies because they lack our aimed outcome. Ultimately, 11 studies were analyzed (Figure 1) (23–33).

### 3.2. Basic characteristics and quality assessment

We extracted the data on the first author, country, publication year, experiment animal number and situation, model establishment situation, group situation, some outcomes and web link. We included nine studies from China (23, 24, 26, 28–33), one from Saudi Arabia (25) and one from Iran (27). A total of 158 stem cell–treated animals and 155 POI model animals were included. Eight studies infused hUCMSC by tail vein (23, 24, 27–31, 33), whereas four injected hUCMSC directly into the ovary (25, 26, 29, 32); in addition, one study compared the effects of these two methods (29). Stem cell concentrations varied, ranging from 1 × 10^5^ to 5 × 10^6^. However, the concentration units in some studies were unclear; thus, we only conducted a subgroup analysis by stem cell concentration in hormone analyses. Transplantation time also varied. Some studies set a series of observation time to better show the effect of hUCMSC. To simplify our analysis, we only chose the data at the end of the study for our meta-analysis (Tables 1, 2). In order to identify the effect of different transplantation time, we conducted a subgroup analysis as well.

**Table 1:** Characteristics of the included studies.

**First author**	**Country**	**Publication year**	**Experiment animal**	**Total animal numbers**	**Animal age**	**Model establishment**	**Establishment time**	**Transplantation time**	**Transplantation route**	Available outcome^*^	**Web link**
Jian Shen	China	2020	BALB/c mice	110	7–8 weeks old	Cyclophosphamide	14 d	60 d	tail vein	Estrous cycle; Follicle number; E_2_; FSH	https://www.wjgnet.com/1948-0210/full/v12/i4/277.htm
He Jie	China	2021	BALB/C female mice	30	7 to 8 weeks	Cyclophosphamide + baixioan	1 d	15 d	tail vein	E_2_; FSH; AMH; LH; Follicle number	https://cellmolbiol.org/index.php/CMB/article/view/4071
Amr K. Elfayomy	Saudi Arabia	2016	albino Wistar rats	95	-	Paclitaxel	1 d	6 wk	*in situ*	Follicle number; E_2_; FSH	https://www.sciencedirect.com/science/article/pii/S0040816616300246?via%3Dihub
Xunyi Zhang	China	2020	SD rats	80	6–8 weeks	pZP3 suspension	1 d	20 d	*in situ*	Estrous cycle; E_2_; FSH; LH; Follicle number	https://www.tandfonline.com/doi/full/10.1080/09513590.2021.1878133
Ladan Jalalie	Iran	2021	C57BL/6 mice	30	6–8-week-old	Cyclophosphamide	15 d	1 wk	tail vein	Follicle number; FSH; E_2_	https://www.sciencedirect.com/science/article/abs/pii/S0065128120301574?via%3Dihub
Taoran Deng	China	2021	C57BL/6 mice	27	6–7 weeks old	Cyclophosphamide and busulfan	1 d	2 wk	tail vein	Estrous cycle; Follicle number; E_2_; FSH	https://link.springer.com/article/10.1007/s43032-021-00499-1
Dan Song	China	2016	Wistar rats	40	8 weeks old	Cyclophosphamide	1 d	6 wk	tail vein+*in situ*	E_2_; FSH; AMH; Follicle number	https://www.hindawi.com/journals/bmri/2016/2517514/
Zhe Wang	China	2020	SD rats	120	8 weeks old	Ovarian antigen	30 d	2 wk	tail vein	Estrous cycle; Follicle number	https://www.hindawi.com/journals/sci/2020/3249495/
Shufang Wang	China	2013	CD1 (ICR) mice	45	-	Cyclophosphamide	15 d	1 wk	tail vein	Follicle number	https://www.hindawi.com/journals/bmri/2013/690491/
Yanjun Yang	China	2019	C57BL/6 mice	24	6 weeks old	Cyclophosphamide	15 d	4 wk	*in situ*	Estrous cycle; E_2_; FSH; AMH; Follicle number	https://link.springer.com/article/10.1007/s11626-019-00337-4
Qun Zheng	China	2019	SD rats	40	12 weeks old	Cyclophosphamide	15 d	2 wk	tail vein	Estrous cycle; AMH; E_2_; FSH; Follicle number	https://www.hindawi.com/journals/bmri/2019/6539294/

^*^Wang et al. (30), Wang et al. (31), and Zheng et al. (33) lack exact data of serum sex hormone.

**Table 2:** Quality assessment of the included studies.

**Study**	**①**	**②**	**③**	**④**	**⑤**	**⑥**	**⑦**	**⑧**	**⑨**	**⑩**
Shen et al. (23)	No	Yes	Unknown	Unknown	Unknown	Unknown	Yes	Yes	Yes	Yes
Jie et al. (24)	Unknown	Yes	Unknown	Unknown	Unknown	Unknown	Yes	Yes	Yes	Yes
Elfayomy et al. (25)	Unknown	Yes	Unknown	Unknown	Unknown	Unknown	Yes	Yes	Yes	Yes
Zhang et al. (26)	Unknown	Yes	Unknown	Unknown	Unknown	Unknown	Yes	Yes	Yes	Yes
Jalalie. et al. (27)	Unknown	Yes	Unknown	Unknown	Unknown	Unknown	Yes	Yes	Yes	Yes
Deng. et al. (23)	Unknown	Yes	Unknown	Unknown	Unknown	Unknown	Yes	Yes	Yes	Yes
Song et al. (28)	Unknown	Yes	Unknown	Unknown	Unknown	Unknown	Yes	Yes	Yes	Yes
Wang et al. (29)	Unknown	Yes	Unknown	Unknown	Unknown	Unknown	Yes	Yes	Yes	Yes
Wang et al. (30)	Unknown	Yes	Unknown	Unknown	Unknown	Unknown	Yes	Yes	Yes	Yes
Yang et al. (31)	Unknown	Yes	Unknown	Unknown	Unknown	Unknown	Yes	Yes	Yes	Yes
Zheng et al. (32)	Unknown	Yes	Unknown	Unknown	Unknown	Unknown	Yes	Unknown	Yes	Yes

SYRCLE animal experiment bias risk assessment form: ① Distribution sequence production is sufficient ② Baselines of groups are the same ③ Distribution concealing is sufficient ④ Experiment animals are randomly fed ⑤ Blinding to researcher ⑥ Randomly select animals to assess result ⑦ Blinding to result evaluator ⑧ No incomplete data ⑨ No selective result report ⑩ No other biases.

### 3.3. Analysis of outcomes

#### 3.3.1. Estrous cycle

**Figure 2:**
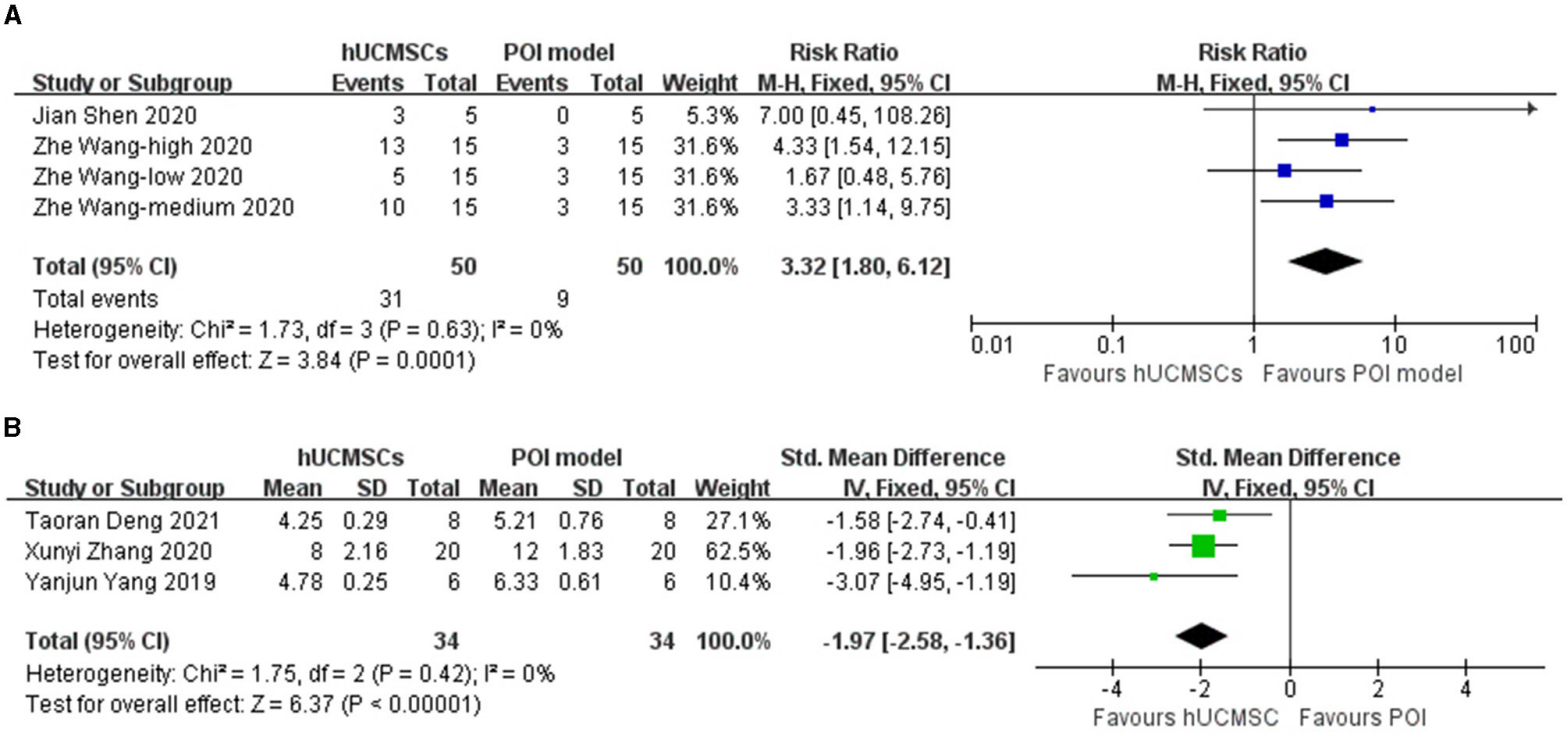
Forest plot of animals' estrous cycle situation in the hUCMSC group versus the POI model group. **(A)** Numerous animals in the hUCMSC group recovered normal estrous cycle compared with those in the POI model group (RR: 3.32, 95% CI: [1.80, 6.12], *I*^2^ = 0%, *P* = 0.0001). **(B)** The cycle length significantly decreased in the hUCMSC group compared with that in the POI model group (SMD: −1.97, 95% CI: [−2.58, −1.36], *I*^2^ = 0%, *P* < 0.00001).

Five studies reported estrous cycle situation (23, 26, 28, 30, 32). Two outcomes were used to divide them into two analyses. Of the five studies, two (23, 30) calculated the proportion of animals with normal estrous cycle. Based on different cell concentrations, four groups were included in one analysis. The three other studies (26, 28, 32) measured the length of the estrous cycle in animals. Results showed that hUCMSC significantly improved the proportion of animals with normal estrous cycle (RR: 3.32, 95% CI: [1.80, 6.12], *I*^2^ = 0%, *P* = 0.0001; Figure 2A) and shortened the estrous cycle length (SMD: −1.97, 95% CI: [−2.58, −1.36], *I*^2^ = 0%, *P* < 0.00001; Figure 2B). Based on the location of stem cell injection, the subgroup analysis showed that estrous cycle improvement is independent of the injection site (Table 3).

**Table 3:** Subgroup analysis results of animals' estrous cycle and serum hormone level.

**Comparison**	**Result**					
Estrous cycle length	Subgroup name	Study number	Included study	SMD [95%CI]	*I* ^2^	*P*
	**Injection location**					
	Tail vein	1	Deng et al. (28)	−1.58 [−2.74, −0.41]	Not applicable	0.008
	*In situ*	2	Zhang et al. (26); Yang et al. (34)	−2.12 [−2.83, −1.41]	13%	< 0.00001
E_2_	Subgroup name	Study number	Included study	SMD [95%CI]	*I* ^2^	P
	**Calculate unit**					
	unknown	1	Shen et al. (23)	9.68 [4.09, 15.27]	Not applicable	0.0007
	pg/mL, ng/L	6	Elfayomy et al. (25); Song et al.–*in situ* (29); Dan Song et al.–tail vein (29); Jie et al. (24); Jalalie et al. (27); Zhang et al. (26)	6.07 [2.84, 9.31]	94%	0.0002
	ng/mL	1	Deng et al. (28)	0.25 [−0.73, 1.24]	Not applicable	0.62
	pmol/L	1	Yang et al. (32)	6.41 [3.86, 8.95]	Not applicable	< 0.00001
	**Injection location**					
	Tail vein	5	Song et al.–tail vein (29); Jie et al. (24); Jalalie et al. (27); Shen et al. (23); Deng et al. (28)	5.32 [1.86, 8.79]	92%	0.003
	*In situ*	4	Elfayomy et al. (25); Song–*in situ* et al. (29); Zhang et al. (26); Yang et al. (32)	6.25 [1.67, 10.82]	95%	0.007
	**Stem cell concentration**				
	1 × 10E5	1	Zhang et al. (26)	0.69 [0.05, 1.33]	Not applicable	0.03
	2 × 10E5	1	Yang et al. (32)	6.41 [3.86, 8.95]	Not applicable	< 0.00001
	1 × 10E6	5	Song–tail vein et al. (29); Jie et al. (24); Jalalie et al. (27); Shen et al. (23); Deng et al. (28)	5.32 [1.86, 8.79]	92%	0.03
	2 × 10E6	2	Elfayomy et al. (25); Song et al.–*in situ* (29)	10.44 [−6.37, 27.24]	97%	0.22
	**Transplantation time**					
	2 weeks or 15 days	5	Elfayomy et al. (25); Song et al.–*in situ* (29); Song et al.–tail vein (29); Shen et al. (23); Deng et al. (28)	2.26 [1.11, 3.42]	61%	0.0001
	4 weeks or 30 days	5	Elfayomy et al. (25); Song et al.–*in situ* (29); Song et al.–tail vein (29); Shen et al. (23); Deng et al. (28)	3.91 [0.96, 6.86]	92%	0.009
	6 weeks or 45 days	4	Elfayomy et al. (25); Song et al.–*in situ* (29); Song et al.–tail vein (29); Shen et al. (23)	7.33 [1.87, 12.78]	93%	0.008
	60 days	1	Shen et al. (23)	9.68 [4.09, 15.27]	Not applicable	0.0007
AMH	Subgroup name	Study number	Included study	SMD [95%CI]	*I* ^2^	P
	**Calculate unit**					
	ng/mL	1	Jie et al. (24)	3.64 [2.11, 5.17]	Not applicable	< 0.00001
	Subgroup name	Study number	Included study	SMD [95%CI]	*I* ^2^	*P*
	pg/mL	3	Song et al.–*in situ* (29); Song et al.–tail vein (29); Yang et al. (32)	1.14 [0.37, 1.92]	0%	0.004
	**Injection location**					
	Tail vein	2	Song et al.–tail vein (29); Jie et al. (24)	2.84 [1.11, 4.57]	51%	0.001
	*In situ*	2	Song et al.–*in situ* (29); Yang et al. (32)	1.00 [0.15, 1.85]	0%	0.02
	**Stem cell concentration**				
	2 × 10E5	1	Yang et al. (32)	0.81 [−0.16, 1.78]	Not applicable	0.1
	1 × 10E6	2	Song et al.–tail vein (29); Jie et al. (24)	2.84 [1.11, 4.57]	51%	0.001
	2 × 10E6	1	Song et al.–*in situ* (29)	1.62 [−0.16, 3.40]	Not applicable	0.07
FSH	Subgroup name	Study number	Included study	SMD [95%CI]	*I* ^2^	P
	**Calculate unit**					
	unknown	1	Shen et al. (23)	−7.28 [−11.56, −3.00]	Not applicable	0.0009
	mIU/mL, U/L, IU/L	7	Elfayomy et al. (25); Song et al.–*in situ* (29); Song et al.–tail vein (29); Jie et al. (24); Jalali et al. (27); Deng et al. (28); Yang et al. (32)	−2.26 [−4.11, −0.41]	92%	0.02
	pg/mL	1	Zhang et al. (26)	−5.27 [−6.64, −3.91]	Not applicable	< 0.00001
	**Injection location**					
	Tail vein	5	Song et al.–tail vein (29); Jie et al. (24); Shen et al. (23); Jalali et al. (27); Deng et al. (28)	−2.51 [−4.19, −0.84]	82%	0.003
	*In situ*	4	Elfayomy et al. (25); Song et al.–*in situ* (29); Zhang et al. (26); Yang et al. (32)	−3.40 [−7.44, 0.65]	96%	0.1
	**Stem cell concentration**				
	1 × 10E5	1	Zhang et al. (26)	−5.27 [−6.64, −3.91]	Not applicable	< 0.00001
	2 × 10E5	1	Yang et al. (32)	1.49 [0.41, 2.56]	Not applicable	0.007
	1 × 10E6	5	Song et al.–tail vein (29); Jie et al. (24); Shen et al. (23); Jalali et al. (27); Deng et al. (28)	−2.51 [−4.19, −0.84]	82%	0.003
	2 × 10E6	2	Elfayomy et al. (25); Song et al.–*in situ* (29)	−4.96 [−11.50, 1.59]	95%	0.14
	**Transplantation time**					
	2 weeks or 15 days	5	Elfayomy et al. (25); Song et al.–*in situ* (29); Song et al.–tail vein (29); Shen et al. (23); Deng et al. (28)	−2.10 [−3.71, −0.50]	79%	0.01
	4 weeks or 30 days	5	Elfayomy et al. (25); Song et al.–*in situ* (29); Song et al.–tail vein (29); Shen et al. (23); Deng et al. (28)	−3.28 [−5.54, −1.01]	86%	0.005
	Subgroup name	Study number	Included study	SMD [95%CI]	*I* ^2^	*P*
	6 weeks or 45 days	4	Elfayomy et al. (25); Song et al.–*in situ* (29); Song et al.–tail vein (29); Shen et al. (23)	−4.03 [−7.09, −0.97]	87%	0.01
	60 days	1	Shen et al. (23)	−7.28 [−11.56, −3.00]	Not applicable	0.0009
LH	Subgroup name	Study number	Included study	SMD [95%CI]	*I* ^2^	P
	**Calculate unit**					
	mIU/mL	1	Jie et al. (24)	−1.46 [−2.47, −0.45]	Not applicable	0.005
	pg/mL	1	Zhang et al. (26)	−2.94 [−3.86, −2.02]	Not applicable	< 0.00001
	**Injection location**					
	Tail vein	1	Jie et al. (24)	−1.46 [−2.47, −0.45]	Not applicable	0.005
	*In situ*	1	Zhang et al. (26)	−2.94 [−3.86, −2.02]	Not applicable	< 0.00001
	**Stem cell concentration**				
	1 × 10E5	1	Zhang et al. (26)	−2.94 [−3.86, −2.02]	Not applicable	< 0.00001
	1 × 10E6	1	Jie et al. (24)	−1.46 [−2.47, −0.45]	Not applicable	0.005

#### 3.3.2. E_2_

**Figure 3:**
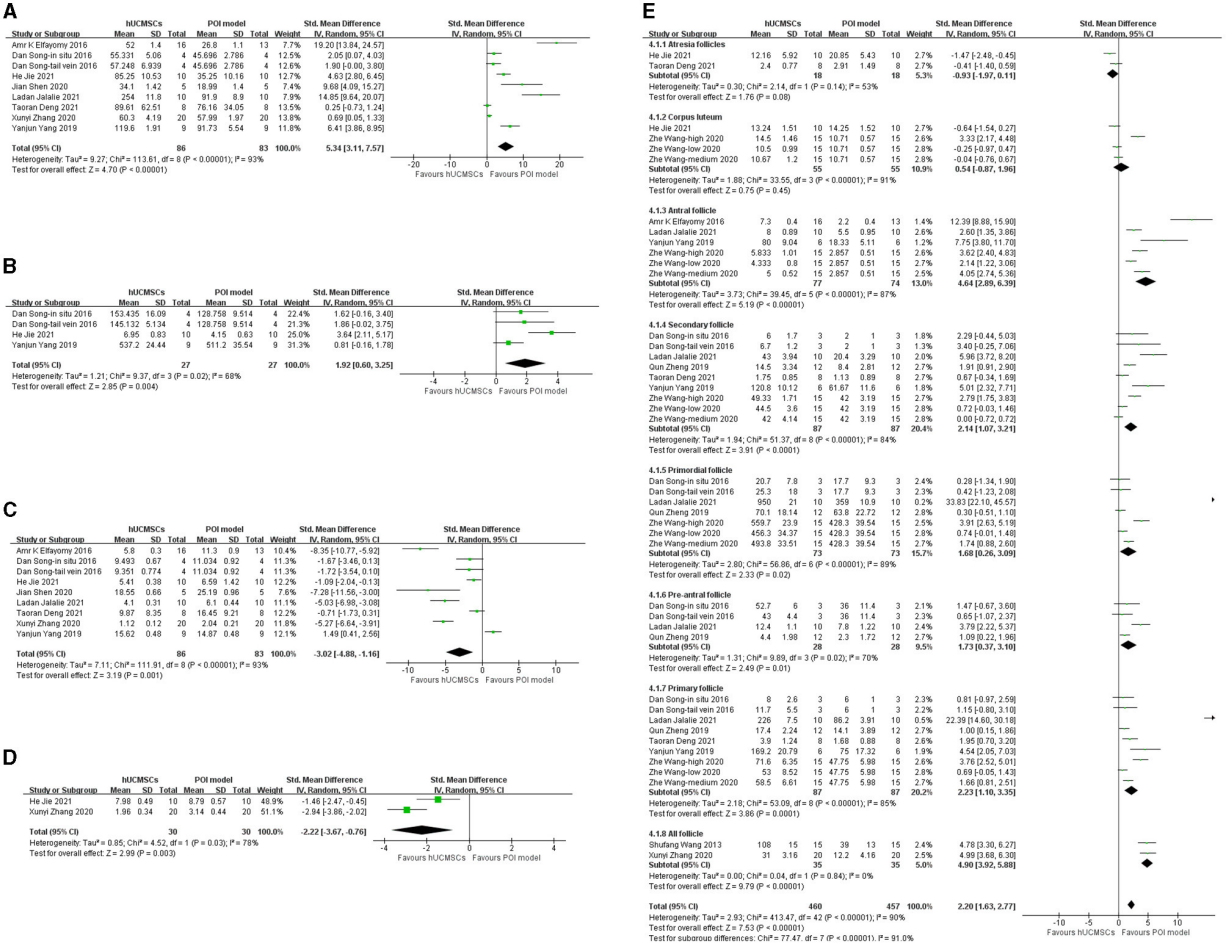
Forest plot of animals' serum hormone level and follicle number in the hUCMSC group vs. the POI model group. **(A)** Serum E_2_ concentrations significantly increased in the hUCMSC group compared with those in the POI model group (SMD: 5.34, 95% CI: [3.11, 7.57], *I*^2^ = 93%, *P* < 0.00001). **(B)** Serum AMH concentrations significantly increased in the hUCMSC group compared with those in the POI model group (SMD: 1.92, 95% CI: [0.60, 3.25], *I*^2^ = 68%, *P* = 0.004). **(C)** Serum FSH concentrations significantly decreased in the hUCMSC group compared with those in the POI model group (SMD: −3.02, 95% CI: [−4.88, −1.16], *I*2 = 93%, *P* = 0.001). **(D)** Serum LH concentrations significantly decreased in the hUCMSC group compared with those in the POI model group (SMD: −2.22, 95% CI: [−3.67, −0.76], *I*^2^ = 78%, *P* = 0.003). **(E)** Ovarian follicle count comparison between the hUCMSC group versus the POI model group. Antral follicles (SMD: 4.64, 95% CI: [2.89, 6.39], *I*^2^ = 87%, *P* < 0.00001), pre-antral follicles (SMD: 1.73, 95% CI: [0.37, 3.10], *I*^2^ = 70%, *P* = 0.01), secondary follicles (SMD: 2.14, 95% CI: [1.07, 3.21], *I*^2^ = 84%, *P* < 0.0001), primary follicle (SMD: 2.23, 95% CI: [1.10, 3.35], *I*^2^ = 85%, *P* = 0.0001), primordial follicle (SMD: 1.68, 95% CI: [0.26, 3.09], *I*^2^ = 89%, *P* = 0.02) and all follicles (SMD: 4.90, 95% CI: [3.92, 5.88], *I*^2^ = 0%, *P* < 0.00001) increased significantly in the hUCMSC group compared with those in the POI model group.

Eight studies reported serum E_2_ levels (23–29, 32). Based on the injection location, nine groups were included in the analysis. Serum E_2_ significantly increased in the hUCMSC group compared with that in the POI model group (SMD: 5.34, 95% CI: [3.11, 7.57], *I*^2^ = 93%, *P* < 0.00001; Figure 3A). We conducted a subgroup analysis according to the statistical units, stem cell injection location and stem cell concentration. Besides, as some included studies compared several transplantation time, we also conducted a subgroup analysis based on it. The elevation of serum E_2_ level was not significant when calculated by ng/mL or when the stem cell concentration was 2 × 10e6. However, the effect of hUCMSC on the serum E_2_ level of animals was independent of the injection location. Significance was observed in all intervention time subgroup, indicating hUCMSC can increase serum E_2_ level at the beginning of 2 weeks (Table 3).

#### 3.3.3. AMH

Three studies reported serum AMH (24, 29, 32). Based on the injection location, four groups were included in the analysis. Serum AMH significantly increased in the hUCMSC group compared with that in the POI model group (SMD: 1.92, 95% CI: [0.60, 3.25], *I*^2^ = 68%, *P* = 0.004; Figure 3B). The subgroup analysis revealed that serum AMH was independent of the statistical units and injection location. However, when the stem cell concentration was 2 × 10e5 and 2 × 10e6, no significant change was observed between the model and hUCMSC groups (Table 3).

#### 3.3.4. FSH

Eight studies reported serum FSH levels (23–29, 32). Based on the injection location, nine groups were included in the analysis. Compared with the POI model group, the hUCMSC group showed a significant reduction in serum FSH (SMD: −3.02, 95% CI: [−4.88, −1.16], *I*^2^ = 93%, *P* = 0.001; Figure 3C). According to the statistical units, stem cell injection location, stem cell concentration and transplantation time, the subgroup analysis showed that no significant change was observed when hUCMSC was injected *in situ* and when the stem cell concentration was 2 × 10e6. However, in most cases, the FSH level decreased significantly after hUCMSC injection. Meanwhile, FSH decreases significantly 2 weeks after hUCMSC injection, indicating the effect of hUCMSC works at the beginning of 2 weeks (Table 3).

#### 3.3.5. LH

Two studies reported serum LH (24, 26). Given that they used different calculation units, injection location and stem cell concentration, we conducted a subgroup analysis. Compared with the POI model group, the hUCMSC group showed a significant decrease in serum LH (SMD: −2.22, 95% CI: [−3.67, −0.76], *I*^2^ = 78%, *P* = 0.003; Figure 3D). Subgroup analysis results showed that the treatment effect on LH concentration was independent of the calculation unit, injection location and stem cell concentration (Table 3).

#### 3.3.6. Follicle number

Ten studies determined the follicle count in animals (24–33). However, considering the various follicle types, we only conducted a subgroup analysis based on the follicle types (Figure 3E). All subgroups, except the atresia follicles (*P* = 0.08) and corpus luteum (*P* = 0.45), showed significant differences. After hUCMSC injection, the antral follicles (SMD: 4.64, 95% CI: [2.89, 6.39], *I*^2^ = 87%, *P* < 0.00001), secondary follicles (SMD: 2.14, 95% CI: [1.07, 3.21], *I*^2^ = 84%, *P* < 0.0001), primordial follicles (SMD: 1.68, 95% CI: [0.26, 3.09], *I*^2^ = 89%, *P* = 0.02), pre-antral follicles (SMD: 1.73, 95% CI: [0.37, 3.10], *I*^2^ = 70%, *P* = 0.01), primary follicles (SMD: 2.23, 95% CI: [1.10, 3.35], *I*^2^ = 85%, *P* = 0.0001) and all follicles (SMD: 4.90, 95% CI: [3.92, 5.88], *I*^2^ = 0%, *P* < 0.00001) significantly increased compared with those in the POI model group.

### 3.4. Sensitivity analysis

By picking out studies one by one, we conducted a sensitivity analysis on five outcomes of the estrous cycle, E_2_, FSH, AMH and LH. Results of the estrous cycle, E_2_, FSH and LH were stable, but after picking out Jie et al. (24) or Yang et al. (32), the heterogeneity of the AMH outcome reduced significantly (Jie et al.: *I*^2^ = 0%, *P* = 0.53; Yang et al.: *I*^2^ = 43%, *P* = 0.17). The data unit, transplantation time and study animals may explain the heterogeneity; larger data are needed to determine the origin (Table 4).

**Table 4:** Sensitivity analysis of the results.

**Outcome**	**Excluded study**	**Number of observation**	***I*** **^2^**	**SMD/RR [95%CI]**	**P value of overall effect**
Normal estrous cycle proportion	–	4	0%	3.32 [1.80, 6.12]	0.0001
	Shen et al. (23)	3	0%	3.11 [1.66, 5.82]	0.0004
	Wang–High et al. (30)	3	0%	2.85 [1.32, 6.13]	0.008
	Wang–Low et al. (30)	3	0%	4.08 [1.99, 8.37]	0.0001
	Wang–Medium et al. (30)	3	0%	3.31 [1.57, 6.97]	0.002
Duration of estrous cycle	–	3	0%	−1.97 [−2.58, −1.36]	< 0.00001
	Zhang et al. (18)	2	43%	−1.99 [−2.98, −1.00]	< 0.0001
	Yang et al. (32)	2	0%	−1.84 [−2.48, −1.20]	< 0.00001
	Deng et al. (28)	2	13%	−2.12 [−2.83, −1.41]	< 0.00001
E_2_	–	9	93%	5.34 [3.11, 7.57]	< 0.00001
	Shen et al. (23)	8	93%	4.95 [2.69, 7.20]	< 0.0001
	Elfayomy et al. (25)	8	90%	3.89 [2.00, 5.79]	< 0.0001
	Song–*in situ* et al. (29)	8	94%	5.93 [3.42, 8.44]	< 0.00001
	Song–tail vein et al. (29)	8	94%	5.96 [3.44, 8.48]	< 0.00001
	Jie et al. (24)	8	93%	5.47 [3.06, 7.89]	< 0.00001
	Jalalie et al. (27)	8	92%	4.35 [2.26, 6.44]	< 0.0001
	Zhang et al. (26)	8	93%	6.51 [3.48, 9.54]	< 0.0001
	Deng et al. (28)	8	93%	6.49 [3.60, 9.38]	< 0.0001
	Yang et al. (32)	8	93%	5.13 [2.83, 7.43]	< 0.0001
FSH	–	9	93%	−3.02 [−4.88, −1.16]	0.001
	Shen et al. (23)	8	93%	−2.67 [−4.57, −0.77]	0.006
	Elfayomy et al. (25)	8	91%	−2.35 [−4.09, −0.62]	0.008
	Song–*in situ* et al. (29)	8	94%	−3.22 [−5.29, −1.15]	0.002
	Song–tail vein et al. (29)	8	94%	−3.21 [−5.27, −1.15]	0.002
	Jie et al. (24)	8	94%	−3.35 [−5.61, −1.09]	0.004
	Jalalie et al. (27)	8	93%	−2.76 [−4.71, −0.82]	0.005
	Deng et al. (28)	8	93%	−3.39 [−5.60, −1.18]	0.003
	Yang et al. (32)	8	90%	−3.60 [−5.39, −1.81]	< 0.0001
	Zhang et al. (26)	8	91%	−2.68 [−4.51, −0.84]	0.004
AMH	–	4	68%	1.92 [0.60, 3.25]	0.004
	Jie et al. (24)	3	0%	1.14 [0.37, 1.92]	0.004
	Song–*in situ* et al. (29)	3	79%	2.04 [0.25, 3.84]	0.03
	Song–tail vein et al. (29)	3	79%	1.97 [0.21, 3.72]	0.03
	Yang et al. (32)	3	43%	2.46 [1.14, 3.78]	0.0003

### 3.5. Publication bias

**Figure 4:**
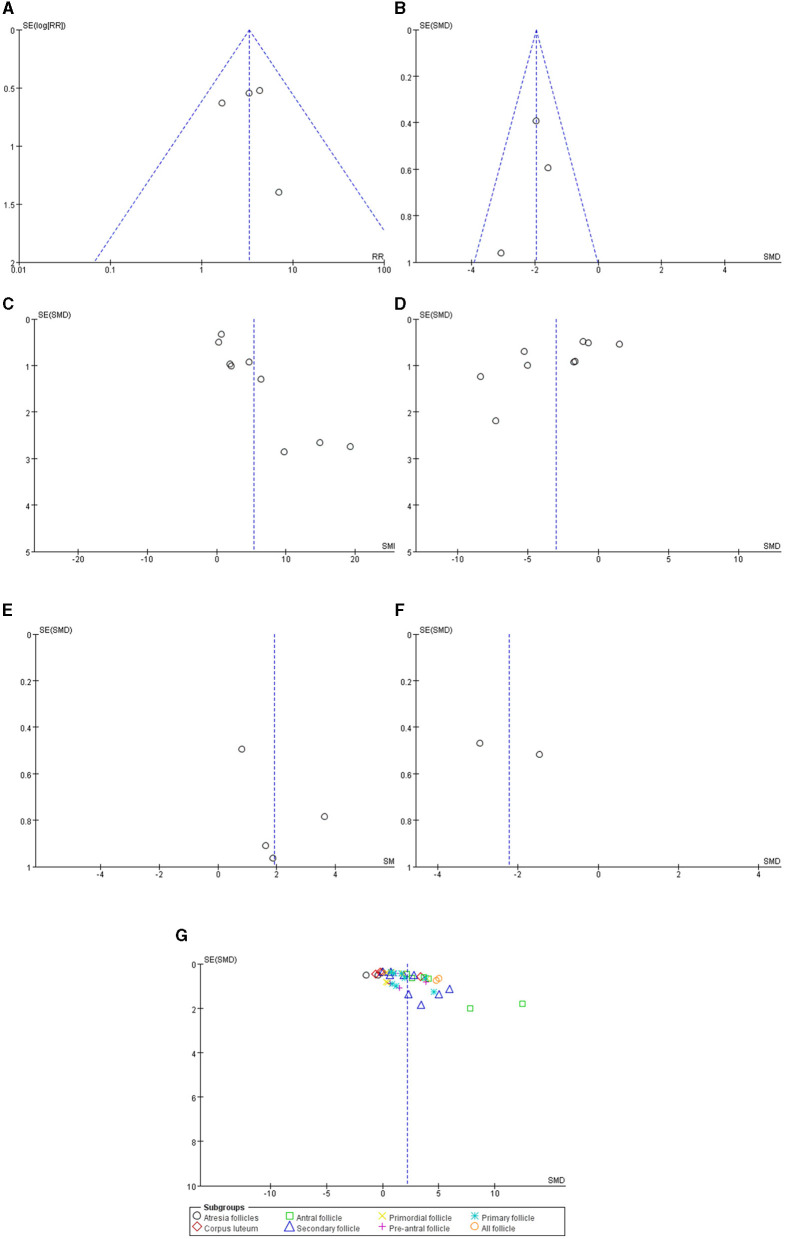
Funnel plots of the **(A)** normal estrous cycle, **(B)** estrous cycle length, **(C)** serum E_2_, **(D)** serum FSH, **(E)** serum AMH, **(F)** serum LH and **(G)** follicle count.

Funnel plots of the normal estrous cycle proportion, E_2_, FSH and follicle number are asymmetric, whereas those of the estrous cycle length, AMH and LH are symmetric. Publication bias likely existed, especially in the results of the estrous cycle, E_2_, FSH and follicle number (Figure 4).

## 4. Discussions

In the year 1942, POI was initially described as a mysterious ailment that perplexed medical practitioners. As the medical community's focus began to shift toward unraveling its elusive nature, POI gradually gained notoriety and its prevalence surged to an alarming 1% (35). Chemical injury is a common method to establish POI model and a main cause of POI clinically apart from genetic disorders (12, 36). But the pathophysiological change of POI is similar regardless of etiology. According to our results, the reduced ovarian function and elevated gonadotropins is reversed by hUCMSC therapy. Follicles are stimulated as well. Therefore, we think the therapeutic effect of hUCMSC is adaptable to all causes. The traditional treatment for POI is HRT, but it only relieves symptoms. The ovarian function remains poor and bring many clinical adverse events about in many patients. In a retrospective cohort study, only 3 of 20 patients achieved pregnancy by assisted reproductive technology (ART) (37). Besides, patients with HRT are more likely to develop sleep problems (38). Given these limitations, stem cell therapy has gained considerable attention recently. Currently, hUCMSC has two other different forms to apply, that is, the microvesicles (34) and extracellular vesicles (39) derived from such cells. Stem cells in the umbilical cord are easy to acquire and do not cause extra donor injury compared with the other stem cell types. Considering their proliferative ability, multidifferentiation and safety, hUCMSC has been researched in the treatment of respiratory (40), cardiovascular (41), liver (42), central nervous system (43, 44) and autoimmune (45) diseases as well as diabetes (46). AMH is crucial in POI diagnosis. In our search on POI, AMH research has gained a large proportion. Physiologically, AMH is secreted by primary ovarian follicles and can negatively regulate the progression of earlier resting follicles into active and progressive ones (47). Considering that AMH secretion slightly varies in menstrual cycle and only healthy follicles secrete it, AMH is considered as a stable marker of ovarian reserve and POI. Our results showed a significant increase of AMH in the hUCMSC group, possibly because of the cytokine secreted by hUCMSC. However, the follicle number increased after stem cell transplantation; the proliferation of healthy follicles may be the cause of the AMH increase. Further research is needed to elucidate the underlying mechanism.

Many factors contribute to the development of infertility. And the gene expressions of different cases are various according to the diseases as a bioinformatic analysis showed (48). As a result, there are only some general treatments of infertility like IVF-ET and artificial insemination. Etiology based therapy of infertility is rare. Infertility therapies are mainly ART, aiming at gaining embryo directly (49). Our meta-analysis confirms folliculogenesis of hUCMSC in animal models, providing an etiology-specific therapy of POI. Apart from efficacy, safety is another important factors under evaluation. Although our result does not cover safety concerns, several phase 1/2 trials have been conducted for hUCMSC in other diseases. General outcomes for safety consideration include immediate infusion related adverse events, blood test like hepatic and renal function and blood cell count, inflammatory cytokine level, hypersensitive, infection, tumorigenesis (50–52). No adverse event is observed in these studies.

Although many studies tried to determine the mechanism of hUCMSC, the specific target remains unclear. Apoptosis regulation was often observed in many animal studies. This result may be derived from some signaling pathways. In a previous study, the expression of CK 8/18, TGF-ß and PCNA increased, while that of CASP-3 decreased (25). Other candidate molecular signals include Bcl-2 (53) and PI3K-Akt (54). Some researchers also hypothesized that angiogenesis can explain the anti-apopotic effect of hUCMSC (32). Further studies should be conducted to determine the exact mechanism and guide the clinical application of hUCMSC. However, hUCMSC can definitely promote ovarian function. Not only AMH but also E_2_, FSH and LH showed significant changes. Estrogen is mainly produced in the ovarian follicle, and LH and FSH play a crucial role. In addition, GCs and their aromatase convert androgen into estrogen (55). Our results proved the therapeutic effect of hUCMSC on E_2_, FSH and LH. We also found some pioneering clinical trials, and their results are optimistic. They investigate antral follicle number and sex hormone of patients to evaluate their ovary function. After UCMSC transplantation, patients showed significant recovery of sex hormone, with decreased level of FSH and increased number of antral follicle (16, 17). Further pregnancy follow up showed that UCMSC transplantation does not affect genetic source of fetus (16). Ovarian volume increases after hUCMSC transplantation with significance, but no significance was observed in collagen scaffold group (17). Nevertheless, compared with animal studies, number of high-quality randomized controlled trials (RCTs) is little. Considering the differences between animals and humans, our meta-analysis result cannot fully support stem cell therapy in POI in human, but can provide a preclinical evidence. However, given that hUCMSC is proven to be effective in POI animals, researchers may pay more attention to RCTs. With abundant and valid RCT evidence, further application of stem cells can be discussed in the future. According to quality assessment table (Table 2), major problems are insufficient randomization and blinding. Thus, it is necessary for researchers to exactly illustrate their randomization settings and blinding measures to prove the function of hUCMSC especially in clinical stage. Traditionally, ovarian follicle number is thought to be fixed and decreases by age (56). So folliculogenesis is traditionally thought as an irreversible procedure. Besides, traditional therapy such as HRT can only relieve symptoms. Fertility preservation is indeed hard to achieve. But recently, the discovery of stem cell in ovary gives a new hope for ovarian regeneration. Germline stem cell (GSC) is identified and isolated from human ovarian cortex. The property of isolated stem cell is proved to be stable after cell culture. *DDX4, OCT4. IFITM3* and *BLIMP-1* are confirmed to be expressed by the GSC (57). Moreover, it can promote ovarian function recovery in sterile animals and achieve pregnancy (58). As our result shows a recovery of ovarian follicle after hUCMSC transplantation. Given the genetic origin of offspring is not from hUCMSC donor as clinical trial proves (16), new experiments can pay some attentions to the effect of hUCMSC on ovarian GSC to explore the mechanism. Many genes can regulate folliculogenesis. Genes, cells or molecules such as *SP1, mTOR, Ube2i, YAP1, C1QTNF3, GPR173*, ovarian fat pad factors, α*-SNAP*, CD11c^+^ cells, M1 MΦs and DCs all play a role in folliculogenesis (59). Some studies have tried to find the association between these genes and the hUCMSC treatment effect. For example, Lu Xueyan et al. found that hUCMSC can inhibit the autophagy of theca-interstitial cells via the AMPK/mTOR signaling pathway (60). Depending on our results, folliculogenesis is a promising direction. Considering that folliculogenesis is tightly connected to anti-apoptosis in GCs (61), some cytokines that can affect GCs may be the target of future mechanism research.

Finally, this study has some limitations that should not be ignored. Clinical study of hUCMSC is rare, and this is also one reason for us to conduct this meta-analysis. Preclinical study is an important part of scientific experiment. Because of limited number of clinical data, it is not the optimal time to conduct a clinical meta-analysis. The meta-analysis of preclinical animal study can promote the development of clinical trails. The relatively insufficient included study, medium quality, high diversity and heterogeneity restrict the application of conclusion. Though random effects model was applied in the analysis, the impact cannot be fully eliminated. All included studies are of medium-quality studies, and higher-quality studies are needed in future research. Due to the limited number of studies, publication bias likely exists in this meta-analysis. However, according to current results, hUCMSC is able to recover ovarian function of POI animals. The result is not affected by limitation. The mentioned limitation should be considered when our conclusion serves as an evidence for clinical study. Thus, we hold a conservative but optimistic view and think more studies are needed in the future to further support the results. Considering the characteristic table, we can observe that a standard animal study procedure has not been formed yet. Future research may focus on a suitable stem cell concentration and transplantation time to eliminate heterogeneity.

## 5. Conclusions

The transplantation of hUCMSC has the potential to restore the estrous cycle, increase E_2_ and AMH levels, decrease FSH and LH levels, and promote folliculogenesis in female rodent models. The results strongly support the use of this therapeutic strategy with a promising outlook. It is important to evaluate the safety and effectiveness of hUCMSC in clinical trials. Randomized controlled trials should also be approached with caution, and safety and adverse effects of hUCMSC should be thoroughly examined in future studies.

## Data availability statement

The original contributions presented in the study are included in the article/supplementary material, further inquiries can be directed to the corresponding authors.

## Author contributions

XW and TL conducted study collection, indentification, data extraction, and statistical disposal. XW drafted the manuscript. TL polished the manuscript. XB, YZ, and MZ collected the relevant references and participated in the discussion. LW designed this meta-analysis and revised the manuscript. All authors contributed to this manuscript. All authors read and approved the final manuscript.
